# Hydroxychloroquine is associated with impaired interferon-alpha and tumor necrosis factor-alpha production by plasmacytoid dendritic cells in systemic lupus erythematosus

**DOI:** 10.1186/ar3895

**Published:** 2012-06-27

**Authors:** Karim Sacre, Lindsey A Criswell, Joseph M McCune

**Affiliations:** 1 Department of Medicine, Division of Experimental Medicine, San Francisco General Hospital, University of California San Francisco, 1001 Potrero Avenue, San Francisco, CA 94110, USA; 2 Department of Medicine, Division of Internal Medicine, APHP, Bichat-Claude Bernard Hospital, Paris Diderot University, 46 rue Henri Huchard, Paris, 75018 France; 3 Department of Medicine, Rosalind Russell Medical Research Center for Arthritis, University of California San Francisco, 374 Parnassus Avenue, San Francisco, CA 94117, USA

## Abstract

**Introduction:**

Plasmacytoid dendritic cells (pDCs) constitutively express two members of the Toll-like receptor (TLR) family, TLR-9 and TLR-7, through which they can be stimulated to produce high levels of interferon (IFN)-α, a key mediator of the pathogenesis of systemic lupus erythematosus (SLE). Given the known efficacy of hydroxychloroquine (HCQ) in the treatment of SLE, we examined its ability to inhibit such pDC function *in vivo*.

**Methods:**

Peripheral blood mononuclear cells (PBMCs) from SLE subjects treated or not with HCQ and from healthy controls were stimulated with the TLR-9 agonist, CpG oligodeoxynucleotides (CpG-A ODN)-2216, and the TLR-7 agonist, imiquimod. The proportion of monocytes, B cells, myeloid dendritic cells, pDCs, and natural killer (NK) cells producing IFN-α and tumor necrosis factor alpha (TNF-α) was then analyzed by multiparameter flow cytometry.

**Results:**

After TLR-9/7 stimulation in both SLE and healthy subjects, significant production of IFN-α and TNF-α was only observed in pDCs. TLR-7 and TLR-9 induced IFN-α and TNF-α production by pDCs from subjects with SLE was decreased relative to that found in controls (TLR-9/IFN-α, *P *< 0.0001; TLR-9/TNF-α *P *< 0.0001; TLR-7/TNF-α *P *= 0.01). TLR-9 and TLR-7 induced IFN-α and TNF-α production by pDCs was severely impaired in 36% (TLR-9) and 33% (TLR-7) of SLE subjects. In almost all cases, these subjects were being treated with HCQ (HCQ vs. no HCQ: impaired TLR-9/IFN-α, *P *= 0.0003; impaired TLR-7/IFN-α, *P *= 0.07; impaired TLR-9/TNF-α, *P *< 0.009; impaired TLR-7/TNF-α, *P *< 0.01).

**Conclusions:**

Treatment with HCQ is associated with impaired ability of pDCs from subjects with SLE to produce IFN-α and TNF-α upon stimulation with TLR-9 and TLR-7 agonists.

## Introduction

A growing body of evidence indicates that type I interferons, such as interferon-α (IFN-α), play a pivotal role in the etiology and pathogenesis of systemic lupus erythematosus (SLE), and single-nucleotide polymorphisms in several key molecules important for IFN-α production and action are associated with SLE [1,2]. Moreover, some of these type I IFN pathway polymorphisms have been shown to impact IFN-α levels and responsiveness in SLE patients *in vivo *[3].

Plasmacytoid dendritic cells (pDCs) have been shown to be the major source of IFN-α production in the peripheral blood [4] and within lymph nodes [5], and these cells produce IFN-α after stimulation across TLR-7 and/or TLR-9 [6-8]. pDCs have also been implicated as key mediators of pathogenesis in SLE [9,10]. However, a number of studies have shown that SLE patients have circulating pDCs that are reduced in number and/or are dysfunctional [11-13]. Since different cell types are known to produce type I IFN in small quantities after microbial challenge [4], these observations raise the possibility that other circulating cells (for example, those involved in the innate immune system, such as monocytes or myeloid dendritic cells, and/or those expressing TLR-9 and/or TLR-7, such as B cells) are the source of IFN-α production in SLE.

Antimalarial agents, such as quinine, have long been used in the treatment of SLE, first (in 1894) in the context of cutaneous lupus and then, as hydroxychloroquine (HCQ), in the context of SLE [14-17]. In a randomized, double-blind, placebo-controlled study, SLE patients treated with HCQ had fewer disease flares and severe disease exacerbations compared to those receiving a placebo [18]. Despite some uncertainty regarding the exact mechanism(s) underlying their various effects, the principal mechanism of action of agents such as HCQ relates to their ability to increase the intracytoplasmic pH and to thereby prevent acidification and maturation of endosomes [19,20]. IFN-α in SLE patients can be produced by pDCs in response to continuous stimulation by circulating immune complexes [9] that are internalized by CD32 (FcγRIIA), with subsequent detection of DNA and RNA by endosomal TLR-9 and TLR-7 in pDCs [10,21]. HCQ would predictably block TLR-9/7 stimulation [22,23] and thus play a beneficial role in the treatment of SLE. Importantly, HCQ has been shown to inhibit the production of IFN-α in pDCs *in vitro*, either after induction by DNA-containing immune complexes [10] or upon stimulation with TLR-9 agonists [13]. It is not clear, however, whether the same effect occurs *in vivo*, that is, in the setting of SLE patients treated with HCQ.

In the current study, we have addressed the above questions directly in a cohort of patients with SLE, treated or not with HCQ, to determine the predominant circulating cell subpopulation capable of IFN-α production and the extent to which such production is inhibited by HCQ *in vivo*.

## Materials and methods

### Patients studied

SLE subjects were recruited from the University of California, San Francisco (UCSF) Lupus Genetics Project collection [1,24]. From this cohort, we recruited 39 individuals of European ancestry who fulfilled at least four of the American College of Rheumatology criteria for SLE [25]. Disease activity was assessed using the Systemic Lupus Activity Questionnaire (SLAQ) [26]. Subjects with the following criteria were excluded: acute infection or vaccination within the prior eight weeks, ongoing treatment with chemotherapy (including cyclophosphamide) or radiotherapy, or active viral hepatitis. Ten healthy gender-matched blood donors served as controls. The University of California, San Francisco Committee on Human Research approved the study and all subjects provided written informed consent.

### Preparation of PBMCs

Blood samples obtained from SLE and healthy control subjects were collected into ethylenediaminetetraacetic acid (EDTA) and peripheral blood mononuclear cells (PBMCs) were prepared using a ficoll hypaque gradient. Cells were washed in phosphate buffered saline (PBS) and suspended in RPMI 1640 medium supplemented with penicillin, streptomycin, and L-glutamine.

### TLR stimulation

PBMCs (10^6^) from SLE and control subjects were cultured with the TLR-7 agonist, imiquimod, (5 μg/ml; InvivoGen, San Diego, CA, USA), the TLR-9 agonist, CpG-A ODN 2216 (5 μM; InvivoGen), the TLR-4 agonist, lipopolysaccharide (0.05 μg/ml; InvivoGen), and media for five hours at 37°C in 5% CO_2_. Brefeldin A (GolgiPlug, BD Pharmingen, San Diego, CA, USA) was added during the final three hours of stimulation to block cytokine secretion.

### Flow cytometry

The panels of antibodies used for phenotypic and intracellular cytokine detection are described in Additional file 1, Table S1. Cytokine detection and phenotyping were performed by sequential cell surface and intracellular staining, following the manufacturer's instructions. Potential Fc receptors were blocked by incubating PBMCs with mouse serum prior to the addition of specific mouse anti-human antibodies. Fluorescence activated cell sorting (FACS) analysis was performed on a four-laser BD LSR-II flow cytometer, and data were analyzed using FlowJo software v9-3 (Treestar, Ashland, OR, USA) and transferred into analysis and graphic software including Excel (Windows, Seattle, WA, USA) and/or GraphPad Prism5 (La Jolla, CA, USA). All analyses were carried out without knowledge of the subject's clinical status, including treatment. The strategy used to gate the different subsets of PBMCs is shown in Additional file 1, Figure S1.

### Statistical analyses

Exact nonparametric two-tailed tests were used. The Kruskal-Wallis One-Way Analysis of Variance on Ranks (ANOVA) or the Mann-Whitney tests were used to compare continuous variables. The Dunn's multiple comparison test was used for statistical correction of multiple comparisons. The Fisher's exact test was used to compare dichotomous variables. The Spearman rank correlation test was used to determine correlations between variables, with r being the Spearman correlation coefficient. Statistical analysis was performed with GraphPad Prism 5.01 software. *P*-values of < 0.05 were considered statistically significant.

## Results

### Characteristics of subjects studied

Table 1 outlines the characteristics of the 39 SLE subjects and 10 healthy controls analyzed in this study. SLE subjects were classified into two groups according to treatment regimen: one group of 25 subjects received hydroxychloroquine (HCQ) and another group of 14 subjects did not. Among the 25 subjects treated with HCQ, 18 received 400 mg/day, 1 received 300 mg/day, and 6 received 200 mg/day. The mean age of the SLE subjects was 53 years and 36 (92%) were female. All subjects were of self-described European descent. The mean SLAQ score, defining lupus disease activity, was 10.4 (± 8.4). Twelve of the SLE subjects (30.8%) were receiving prednisone at a daily dose that was less that 10 mg/day. Eight (20.5%) were also treated with immunosuppressive medication, for example, cyclosporine (*n *= 2), mycophenolate mofetil (*n *= 3), methotrexate (*n *= 2), or azathioprine (*n *= 1). Compared to controls, SLE subjects were older (53 ± 12.5 vs. 30 ± 4.3 years old, *P *< 0.0001). Compared to SLE subjects being treated with HCQ, SLE subjects not receiving HCQ were similar in terms of age, gender, disease activity and other current treatment.

**Table 1:** Characteristics of SLE patients and healthy controls studied

	Healthy	SLE subjects		
	(*n *= 10)	All (*n *= 39)	HCQ (*n *= 25)	No HCQ (*n *= 14)
Age, years (SD)	30 (4.3)^1^	53 (12.5)^1^	50.4 (12.7)	57.6 (11.2)
Female, n (%)	8 (80%)	36 (92%)	22 (88%)	14 (100%)
European ancestry, n (%)	10 (100%)	39 (100%)	25 (100%)	14 (100%)
Years since SLE diagnosis (SD)		19.2 (9.7)	17.2 (9.4)	22.7 (9.6)
SLE activity (SLAQ)		10.4 (8.4)	11.8 (8.9)	8.1 (7.3)
Treatment				
prednisone, n (%)		12 (30.8%)	9 (36%)	3 (21.4%)
immunosuppressant, n (%)		8 (20.5)	5 (20%)	3 (21.4%)

1, *P *< 0.0001. HCQ, hydroxycholoroquine; SD, standard deviation; SLAQ, Systemic Lupus Activity Questionnaire; SLE, systemic lupus erythematosus

### Plasmacytoid dendritic cells are responsible for IFN-α and TNF-α production upon TLR-9/7 stimulation in both SLE and healthy subjects

**Figure 1:**
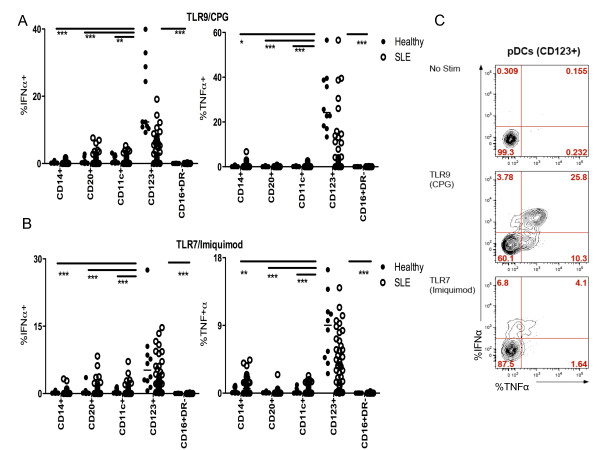
**Plasmacytoid dendritic cells are responsible for IFN-α and TNF-α production upon TLR-9/7 stimulation**. Flow cytometric analysis of IFN-α (left) and TNF-α (right) production after TLR-9 (**A**) or TLR-7 (**B**) stimulation of monocytes (CD14+), B cells (CD20+), mDCs (CD11c+), pDCs (CD123+), and NK cells (CD16+) found in the PBMCs of SLE patients (*n *= 39, white circles) and healthy (*n *= 10, black circles) (see supplementary Figure S1 for details on phenotypic analysis). The lines represent the median IFN-α/TNF-α production by subsets of PBMCs. (C) Flow cytometric analysis of IFN-α and TNF-α production after media (No Stim, top), TLR-9 (middle) and TLR-7 (bottom) stimulation of plasmacytoid dendritic cells (pDCs), (HLA-DR+CD14-CD16-CD20-) CD123+ in one representative subject. *, refers to *P *< 0.05, ** refers to *P *< 0.005, *** refers to *P *< 0.0001.

We first investigated the production of IFN-α and TNF-α by PBMCs upon TLR-9 or TLR-7 stimulation in SLE and healthy subjects. PBMCs from subjects with SLE and healthy controls were stimulated *in vitro *for five hours using the TLR-9 ligand, CpG-A ODN 2216, and the TLR-7 ligand, imiquimod R837. IFN-α and TNF-α production in monocytes (HLA-DR+CD14+), B cells (HLA-DR+CD14-CD16-CD20+), myeloid dendritic cells (mDCs, HLA-DR+CD14-CD16-CD20-CD11c+), pDCs (HLA-DR+CD14-CD16-CD20-CD123+), and natural killer cells (NK cells, HLA-DR-CD16+) was determined using multiparameter flow cytometry (see Additional file 1, Figure S1). In SLE and healthy subjects overall, TLR-9 stimulation induced the production of IFN-α and TNF-α in 0.3% (SD ± 0.3) and 0.8% (± 1.2) of monocytes, 1.3% (± 1.8) and 0.2% (0.2) of B cells, 1.2% (± 1.3) and 0.5% (0.6) of mDCs, and 7.5% (± 8.7) and 10.3% (± 13.5) of pDCS (Figure 1A). After stimulation of TLR-7, a similar analysis of SLE and healthy subjects showed induction of IFN-α and TNF-α in 0.4% (± 0.6) and 0.9% (± 1.1) of monocytes, 1% (± 1.6) and 0.1% (± 0.1) of B cells, 0.8% (± 1.3) and 0.4% (± 0.5) of mDCs, and 4.8% (± 5) and 5.5% (± 4.2) of pDCs (Figure 1B). Of these levels of induction, those observed in pDCs from SLE patients and controls were significantly higher than those observed in the other cell subpopulations. No production of IFN-α or TNF-α was noted in NK cells in either group or with either stimulation. Therefore, among various subpopulations of PBMCs, pDCs represent the major source of IFN-α and TNF-α after TLR-9/7 stimulation in both SLE and healthy subjects (see, for example, Figure 1C).

### pDCs from SLE patients show impaired production of IFN-α and TNF-α after TLR-9/7 stimulation

**Figure 2:**
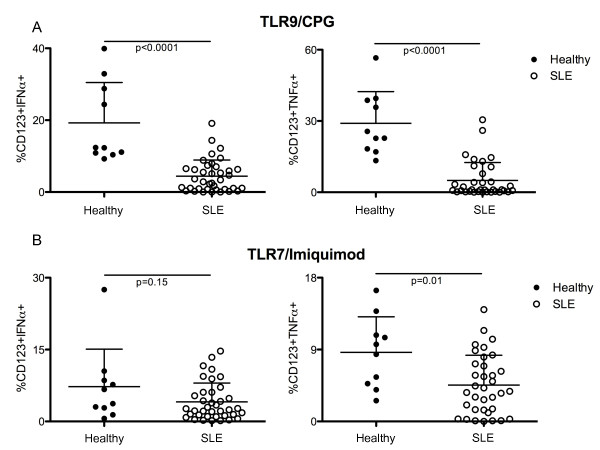
**pDCs from SLE patients show impaired production of IFN-α and TNF-α after TLR-9/7 stimulation**. Comparison of the frequency of circulating pDCs (CD123^+ ^cells) producing IFN-α (left) and TNF-α (right) after TLR-9 (**A**) or TLR-7 (**B**) stimulation between SLE subjects (*n *= 39, white circles) and healthy controls (*n *= 10, black circles). In each case, the lines represent the mean value and the error bars show the standard deviation.

Upon TLR-9 stimulation with CpG, 19.2% (± 3.5) and 29% (± 4) of pDCs from healthy subjects produced IFN-α and TNF-α, respectively. By contrast, only 4.4% (± 0.7) and 8.1% (± 2.1) of pDCs from SLE subjects produced IFN-α (*P *< 0.0001), as compared to healthy controls) and TNF-α (*P *< 0.0001, as compared to healthy controls) (Figure 2A). Likewise, the production of IFN-α and TNF-α by pDCs upon TLR-7 stimulation with imiquimod was decreased in SLE subjects (4% ± 0.6 and 4.4% ± 0.6, respectively) compared to healthy controls (7.2% ± 2.5 and 8.6% ± 1.4, respectively). However, only the difference in the TNF-α production between these two groups was statistically significant (*P *= 0.01) (Figure 2B).

**Figure 3:**
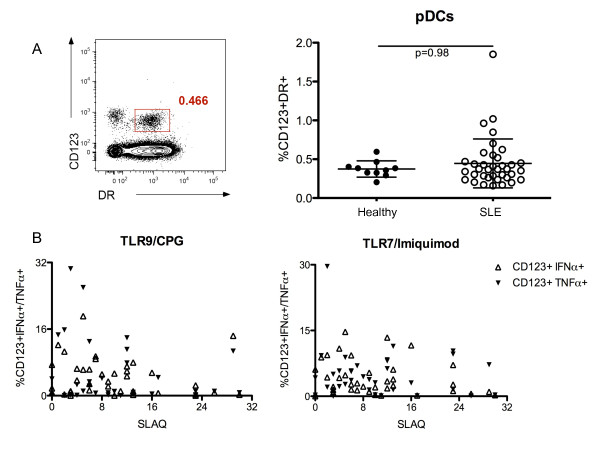
**Frequency of circulating pDCs, pDCs IFN-α+/TNF-α+ after TLR-9/7 stimulation and lupus disease activity**. (**A**) Flow cytometric analysis of pDCs (HLA-DR+CD123+ cells) found in the PBMCs in one representative subject (left). Comparison of the frequency of circulating pDCs found in SLE subjects (white circles) and healthy controls (black circles) (right). (**B**) Absence of statistical correlation between the frequency of CD123+ pDCs IFN-α+ (white triangles) or TNF-α+ (black triangles) after TLR-9 (left) or TLR-7 (right) stimulation and disease activity assessed by using the Systemic Lupus Activity Questionnaire (SLAQ) in SLE patients (TLR-9 stimulation: IFN-α/SLAQ, r = -0.20, *P *= 0.23; TNF-α/SLAQ, r = -0.08, *P *= 0.62) (TLR-7 stimulation: IFN-α/SLAQ, r = -0.18, *P *= 0.28; TNF-α/SLAQ, r = -0.01, *P *= 0.96). In each case, the lines represent the mean value and the error bars show the standard deviation.

To determine if the proportion of pDCs in subjects with SLE was also low (as has been reported [12]), we examined the proportion of pDCs (as defined by the percentage of HLA-DR+, CD123+ cells, relative to all singlet, live cells). At least in this study, the mean percentage of pDCs in SLE subjects (0.46% ± 0.3) did not differ significantly from that observed in healthy controls (0.37% ± 0.1; *P *= 0.98) (Figure 3A). Moreover, the percentage of pDCs did not significantly differ between SLE subjects receiving (0.42% ± 0.33) or not receiving (0.48% ± 0.28) HCQ (*P *= 0.50) (Additional file 1, Figure S2A).

We then examined the relationship between the level of pro-inflammatory cytokines (that is, IFN-α and TNF-α) produced by pDCs and the clinical activity of SLE (as defined using the Systemic Lupus Activity Questionnaire, or SLAQ). No statistical correlation was found between the production of IFN-α and TNF-α upon TLR-9/7 stimulation and the SLAQ scores (Figure 3B). The ability of pDCs to produce IFN-α/TNF-α upon TLR-9/7 stimulation was also not associated with age (age and TLR-9/IFN-α, r = 0.20, *P *= 0.21) (age and TLR-9/TNF-α, r = 0.20, *P *= 0.24) (age and TLR-7/IFN-α, r = 0.12, *P *= 0.45) (age and TLR-7/TNF-α, r = 0.01, *P *= 0.95) (Additional file 1, Figure S2B).

### pDC production of IFN-α and TNF-α upon TLR-9/7 stimulation is dramatically reduced in SLE subjects treated with HCQ

IFN-α and TNF-α production by pDCs upon TLR-9 and TLR-7 stimulation was severely impaired (< 1% of CD123+ IFN-α+/TNF-α+) in 36% (14 subjects) and 33% (13 subjects) of SLE subjects, respectively. Interestingly, all of the 14 SLE subjects with impaired TLR-9 induced IFN-α production and 11 of 13 with impaired TLR-7 induced IFN-α production were being treated with HCQ (impaired TLR-9/IFN-α; HCQ vs no HCQ; *P *= 0.0003) (impaired TLR-7/IFN-α; HCQ vs no HCQ; *P *= 0.07). Likewise, 13 of the 14 SLE subjects with impaired TNF-α production upon TLR-9 stimulation (HCQ vs no HCQ; *P *< 0.009) and 12 of the 13 subjects with impaired TNF-α production upon TLR-7 stimulation (HCQ vs no HCQ; *P *< 0.01) were being treated with HCQ.

**Figure 4:**
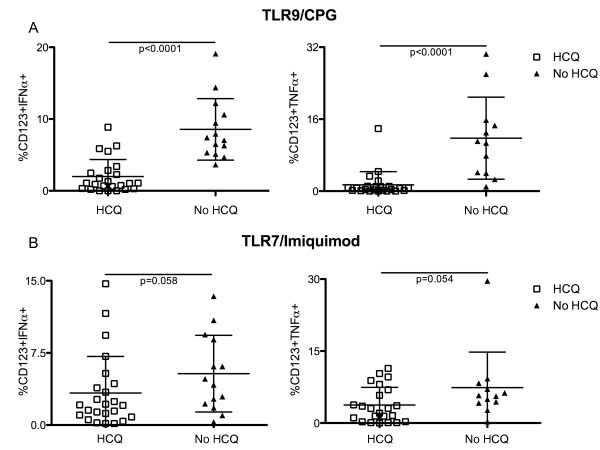
**pDC production of IFN-α/TNF-α upon TLR-9/7 stimulation in SLE subjects treated with HCQ**. Comparison of the frequency of pDCs (CD123^+ ^cells) producing IFN-α (left) and TNF-α (right) after TLR-9 (**A**) or TLR-7 (**B**) stimulation between SLE subjects that were treated (*n *= 25, white squares) or not (*n *= 14, black triangles) with HCQ. In each case, the lines represent the mean value and the error bars show the standard deviation.

**Figure 5:**
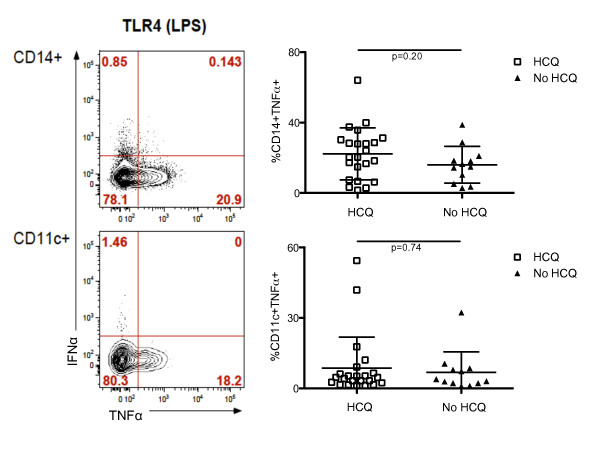
**Monocytes and mDCs production of IFN-α/TNF-α upon TLR-4 stimulation in SLE subjects treated with HCQ**. Flow cytometric analysis (left) of IFN-α and TNF-α production after TLR-4 (lipopolysaccharide, LPS) stimulation of monocytes (CD14+) (top, left) and mDCs, (CD11c+) (bottom) in a representative subject. Comparison of the frequency of monocytes (CD14+) (right top) and mDCs (CD11c+) (right bottom) cells producing TNF-α after TLR-4 stimulation between SLE subjects that were treated (white squares) or not (black triangles) with HCQ. In each case, lines represented the mean value and error bars the standard deviation.

Overall, pDC production of IFN-α and TNF-α upon TLR-9 and TLR-7 stimulation was lower in SLE subjects treated with HCQ (TLR-9: 2% ± 2.2 CD123+IFN-α+; 1.4% ± 2.9 CD123+TNF-α+) (TLR-7: 3.3% ± 3.8 CD123+IFN-α+; 3.7% ± 3.7 CD123+TNF-α+) compared to SLE subjects who were not receiving HCQ (TLR-9: 8.6% ± 4.3 CD123+IFN-α+, *P *< 0.0001; 11.8% ± 9.1 CD123+TNF-α+, *P *< 0.0001) (TLR-7: 5.3% ± 3.9 CD123+IFN-α+, *P *= 0.058; 7.4% ± 7.4 CD123+TNF-α+, *P *= 0.054) (Figure 4). This difference was highly significant (*P *< 0.0001) in the case of TLR-9 stimulation and trended towards significance (*P *< 0.06) in the case of TLR7 stimulation. We did not observe any significant differences in IFN-α/TNF-α production by TLR-9/7-stimulated pDCs between subjects receiving 400 mg/day and 200 mg/day (TLR-9/IFN-α, *P *= 0.35) (TLR-9/TNF-α, *P *= 0.27) (TLR-7/IFN-α, *P *= 0.42) (TLR-7/TNF-α, *P *= 0.13) (Additional file 1, Figure S3). Interestingly, HCQ treatment did not have any effect on the production of TNF-α by monocytes (CD14+) and mDCs (CD11c+) upon TLR-4 stimulation. Indeed, among SLE patients receiving HCQ or not, 22.2% (± 14.7) and 16% (± 10.4) of monocytes, respectively, produced TNF-α after five hours of *in vitro *lipopolysaccharide (LPS) stimulation (*P *= 0.20). In addition, 8.7% (± 13.2) and 6.9% (± 8.6) of mDCs produced TNF-α upon LPS stimulation among SLE patients receiving HCQ or not (*P *= 0.74) (Figure 5).

## Discussion

Ongoing IFN-α production in SLE patients appears to play an important pathogenic role in the autoimmune process and pDCs have a pivotal role as the main producers of IFN-α *in vivo *[9,27]. For this reason, different regimens directed against IFN-α are of high therapeutic benefit in SLE. In this study, we found that pDC production of IFN-α and TNF-α upon TLR-9 or TLR-7 stimulation was markedly reduced in SLE patients treated with HCQ. Although HCQ has been shown to act as a TLR-9 and TLR-7 antagonist *in vitro*, these data demonstrate that it also inhibits TLR-9 and TLR-7 stimulation *in vivo*. Such inhibition is also specific, in that HCQ treatment has little effect on TLR-4-induced production of TNF-α by monocytes and mDCs. Thus, our data provide new insights regarding the mechanism of action of HCQ in SLE. The reduction of TNF-α TLR-9/7-induced pDC production observed with HCQ is interesting from a therapeutic point of view, even if the involvement of TNF-α in the pathogenesis of SLE remains controversial [28]. Interestingly, a recent paper has shown that circulating TNF-α and type I IFN levels are correlated in a large cohort of SLE patients [29]. Finally, similar observations have been made in HCQ-treated HIV-infected "immunologic non-responders" [28], a condition in which chronic exposure to IFN-α may also lead to immune dysfunction [30].

One puzzling and previously reported result is the overall diminished IFN-α production by pDCs in SLE patients compared to controls [11-13]. It has been hypothesized that the reduced ability of pDCs to produce IFN-α may be a consequence of the redistribution of the efficient pDCs into tissues [31] or of the exhaustion of circulating pDCs as a consequence of a high level of stimulation by persistent endogenous IFN-α inducing factors [13]. Although pDCs are probably migrating to tissues and producing IFN- α there, we did not observe any difference in the percentage of circulating pDCs between SLE patients and healthy controls. We did not either observe any association with IFN-α production by *in vitro *stimulated pDCs and SLE disease activity. Kwok *et al. *[13] previously showed that repeated stimulation of pDC with TLR-9 ligand decreased levels of IFN-α production. These authors concluded that the persistent presence of endogenous IFN-α-inducing factors induced TLR tolerance in pDCs of SLE patients. We have now demonstrated that, at least in some patients, such "tolerance" is strongly correlated with the *exogenous *administration of HCQ. In fact, most of the SLE patients in previous studies who showed decreased IFN-α production were receiving HCQ [11-13]. Our comprehensive study of all subsets of PBMCs potentially targeted by TLR-9/7 ligand also excludes the possibility that other circulating mononuclear cells (besides pDCs) may produce IFN-α. It would have been of interest to determine IFN-α levels and the absolute number of pDCs in the circulation, but such measurements are themselves only uncertain surrogates of the IFN-α and pDC levels in tissues, where most of the pathology of SLE plays out. Although conflicting data may result from the use of different methods to isolate and culture the pDCs between studies, our results support a key role for HCQ treatment in explaining the impaired functional ability of pDCs in SLE.

Low serum HCQ levels have been reported to be a marker for and a predictor of SLE exacerbations [32], leading to the question of a possible relationship between blood HCQ concentrations and efficacy. We did not observe any significant differences in IFN-α/TNF-α production by TLR-7/9-stimulated pDCs between subjects receiving 400 mg/day and 200 mg/day. We do not know, however, the extent to which each patient was compliant with treatment and serum HCQ levels were not routinely measured.

Lupus activity is commonly measured by valid and reliable disease activity physician-rated scores, such as the Systemic Lupus Activity Measure (SLAM) [33], the Systemic Lupus Erythematosus Disease Activity Index (SLEDAI) [34], or the British Isles Lupus Assessment Group (BILAG) [35]. All assess cumulative disease activity, including not only parameters that are immunological (for example, the level of antibodies against dsDNA and complement) but also clinical and biological. Practical considerations limited our ability to obtain all of the biological and immunological parameters required to calculate the SLEDAI or BILAG scores.

The SLAQ is a patient-reported assessment of subjective disease activity in SLE that has been shown to correlate strongly (*r *= 0.62) with the SLAM-omitting laboratory items [26] and is considered to be the best measure of self-reported disease activity in the field. It also has the advantage of being the most cost effective way to track disease activity. Using a cutoff score of 13 points on the SLAQ results in a negative predictive value of 74% for clinically important disease activity [26] defined by a SLAM-omitting laboratory items score ≥3 points. In our study, 30 (77%) subjects had a SLAQ score that was less than 13 and the mean SLAQ score was 10.4. We did not observe an association between TLR-9/7 induced IFN-α production by pDCs and SLE disease activity. However, most of the patients studied here had low levels of disease activity and, because of the number of subjects included, our study may have lacked power to fully address this question.

## Conclusions

Despite evidence supporting beneficial effects of HCQ treatment in SLE, few clinical studies have been performed to elucidate the specific mechanisms of action. Our data suggest that HCQ is of therapeutic benefit in SLE because its use is associated with impairment of the *in vivo *production of IFN-α and TNF-α by circulating pDCs upon stimulation of TLR-9 and TLR-7. Possibly its efficacy is limited because it more effectively inhibits TLR9 than TLR7 responses. If so, this would provide impetus for the development of additional TLR-7 and TLR-9 antagonists to use as possible treatment options for SLE.

## Abbreviations

BILAG: British Isles Lupus Assessment Group; FACS: fluorescence-activated cell sorting; HCQ: hydroxychloroquine; IFN: interferon; LPS: lipopolysaccharide; mDCs: myeloid dendritic cells; NK: natural killer; PBMCs: peripheral blood mononuclear cells; pDCs: plasmacytoid dendritic cells; SLAM: Systemic Lupus Activity Measure; SLAQ: Systemic Lupus Activity Questionnaire; SLE: systemic lupus erythematosus; SLEDAI: Systemic Lupus Erythematosus Disease Activity Index; TLR: toll like receptor; TNF: tumor necrosis factor.

## Competing interests

The authors do not have conflicts of interest related to this study.

## Authors' contributions

KS conceived the project. JMM and KS designed and interpreted the experiments, and wrote the manuscript. KS conducted the experiments. LAC selected appropriate subjects for analysis, provided samples and clinical data, and helped to analyze data and to write the paper. All authors have read and approved the manuscript for publication.

## Supplementary Material

Additional file 1 **Table S1. Panel of antibodies used**. Additional file 1, Figure S1. Flow cytometry gating Strategy. Gating strategy by flow cytometry for the detection of monocytes (singlet^1 ^(forward scatter A (FSC-A) × FSC-H diagonal), live (Aqua)-, HLA-DR+, CD14+ cells), NK (singlet, live, HLA-DR-, CD16+ cells), B cells (singlet, live, HLA-DR+, CD14-, CD20+ cells), mDCs (singlet, live, HLA-DR+, CD14- cells, CD20-, CD11c+) and pDCs (singlet, live, HLA-DR+, CD14- cells, CD20-, CD123+). ^1 ^gated singlet live means that all potential doublets were excluded from the analysis. Additional file 1, Figure S2. Frequency of circulating pDCs, pDCs IFN-α+/TNF-α+ after TLR-9/7 stimulation and age. (**A**) Comparison of the frequency of circulating pDCs found in SLE subjects receiving (black circles) or not (black squares) hydroxychloroquine (HCQ). (**B**) Absence of statistical correlation between the frequency of CD123+ pDCs IFN-α+ (white triangles) or TNF-α+ (black triangles) after TLR-9 (left) or TLR-7 (right) stimulation and age. Additional file 1, Figure S3. pDC production of IFN-α/TNF-α upon TLR-9/7 stimulation in SLE subjects treated with HCQ. Comparison of the frequency of pDCs (CD123^+ ^cells) producing IFN-α (left) and TNF-α (right) after TLR-9 (**A**) or TLR-7 (**B**) stimulation between SLE subjects that were receiving 200 mg (HCQ 200 mg, black circles) or 400 mg (HCQ 400 mg, black squares) of hydroxychloroquine (HCQ). Click here for file
