# Supplementary material for: Hydroxychloroquine is associated with impaired interferon-alpha and tumor necrosis factor-alpha production by plasmacytoid dendritic cells in systemic lupus erythematosus
Source: Arthritis Res Ther. 2012 Jun 27;14(3):R155. doi: 10.1186/ar3895 (PMC3446541; doi:10.1186/ar3895)
Supplement: Additional file 1 — Table S1. Panel of antibodies used. Additional file 1, Figure S1. Flow cytometry gating Strategy. Gating strategy by flow cytometry for the detection of monocytes (singlet1 (forward scatter A (FSC-A) × FSC-H diagonal), live (Aqua)-, HLA-DR+, CD14+ cells), NK (singlet, live, HLA-DR-, CD16+ cells), B cells (singlet, live, HLA-DR+, CD14-, CD20+ cells), mDCs (singlet, live, HLA-DR+, CD14- cells, CD20-, CD11c+) and pDCs (singlet, live, HLA-DR+, CD14- cells, CD20-, CD123+). 1 gated singlet live means that all potential doublets were excluded from the analysis. Additional file 1, Figure S2. Frequency of circulating pDCs, pDCs IFN-α+/TNF-α+ after TLR-9/7 stimulation and age. (A) Comparison of the frequency of circulating pDCs found in SLE subjects receiving (black circles) or not (black squares) hydroxychloroquine (HCQ). (B) Absence of statistical correlation between the frequency of CD123+ pDCs IFN-α+ (white triangles) or TNF-α+ (black triangles) after TLR-9 (left) or TLR-7 (right) stimulation and age. Additional file 1, Figure S3. pDC production of IFN-α/TNF-α upon TLR-9/7 stimulation in SLE subjects treated with HCQ. Comparison of the frequency of pDCs (CD123+ cells) producing IFN-α (left) and TNF-α (right) after TLR-9 (A) or TLR-7 (B) stimulation between SLE subjects that were receiving 200 mg (HCQ 200 mg, black circles) or 400 mg (HCQ 400 mg, black squares) of hydroxychloroquine (HCQ). [file ar3895-S1.PDF]

Table S1. Panel of antibodies used

	PBMC subpopulations/ TLR stimulation			
Dye	Antigen	Clone	Manufacture	Dilution
PerCP-Cy5.5	CD123	7G3	BD	12.5
PE-Cy7	TNFα	Mab11	BD	100
PE-TR	CD20	B9E9	Coulter	50
Al700	CD11b	ICRF44	BD	50
APC	IFNα	LT27:295	MACS	100
APC-Cy7	HLA-DR	G46-6	BD	25
PB	CD16	3G8	BD	50
Qdot 605	CD14	929	Invitrogen	1000
Amcyan	Live/Dead		Invitrogen	250

S1

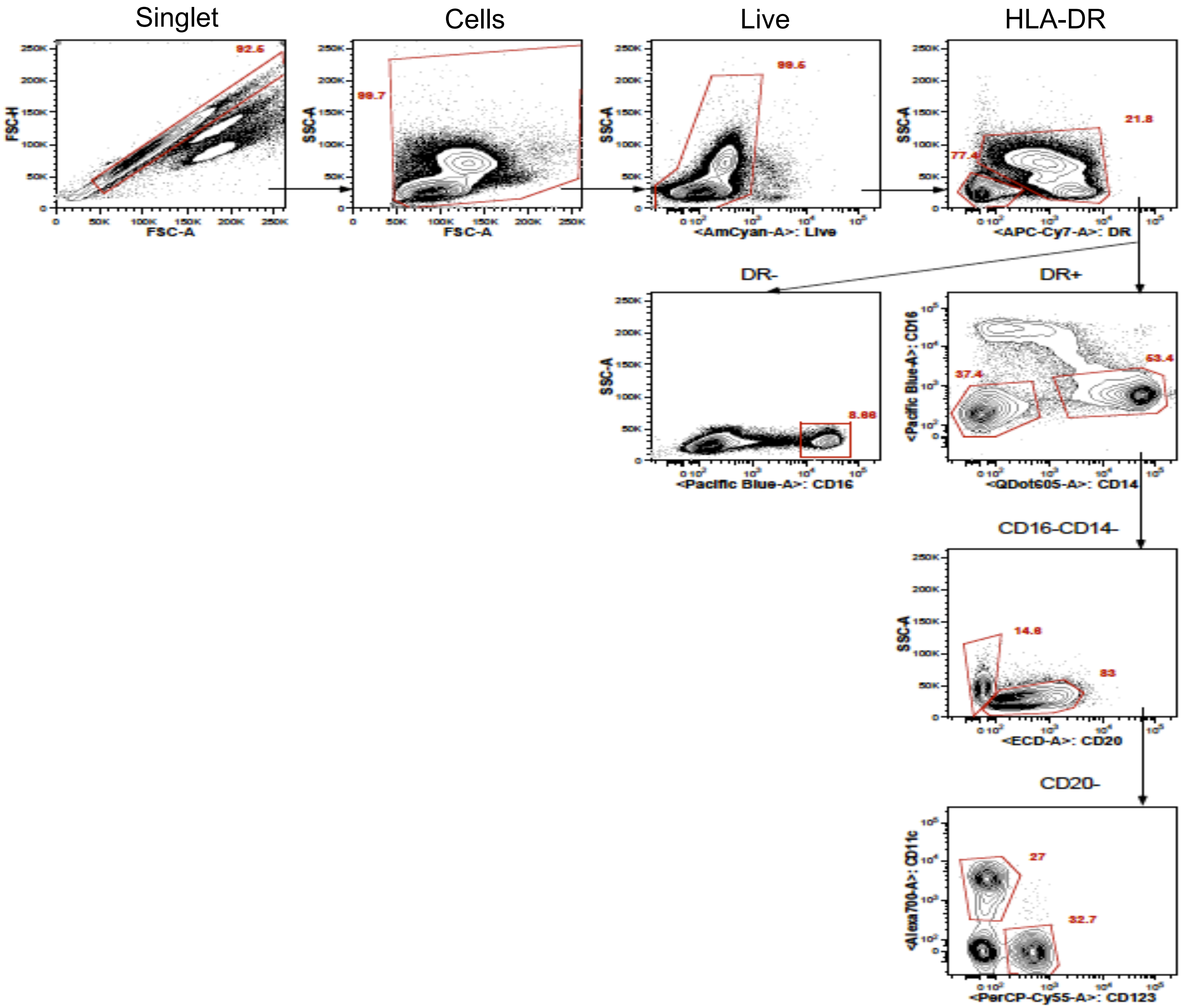
Singlet
Cells
Live
HLA-DR
92.6
FSC-H
FSC-A
99.7
SSC-A
FSC-A
99.6
SSC-A
<AmCyan-A>: Live
21.8
77.4
SSC-A
<APC-Cy7-A>: DR
DR-
DR+
8.86
SSC-A
<Pacific Blue-A>: CD16
37.4
53.4
<Pacific Blue-A>: CD16
<QDot605-A>: CD14
CD16-CD14-
14.6
83
SSC-A
<ECD-A>: CD20
CD20-
27
32.7
<Alexa700-A>: CD11c
<PerCP-Cy55-A>: CD123

S2

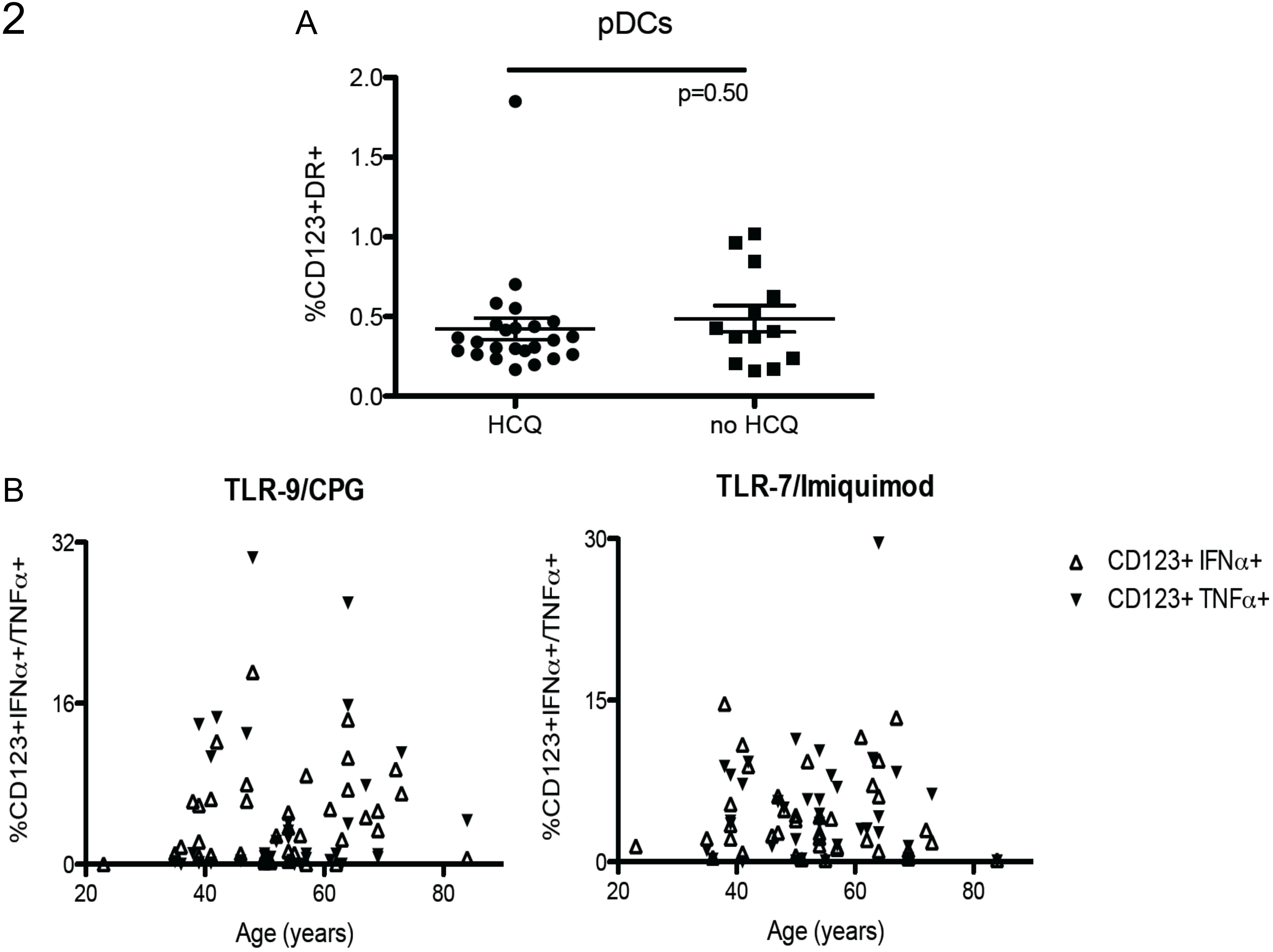
A
pDCs
p=0.50
%CD123+DR+
2.0
1.5
1.0
0.5
0.0
HCQ
no HCQ
B
TLR-9/CPG
%CD123+IFNα+/TNFα+
32
16
0
20
40
60
80
Age (years)
TLR-7/Imiquimod
30
15
CD123+ IFNα+
CD123+ TNFα+

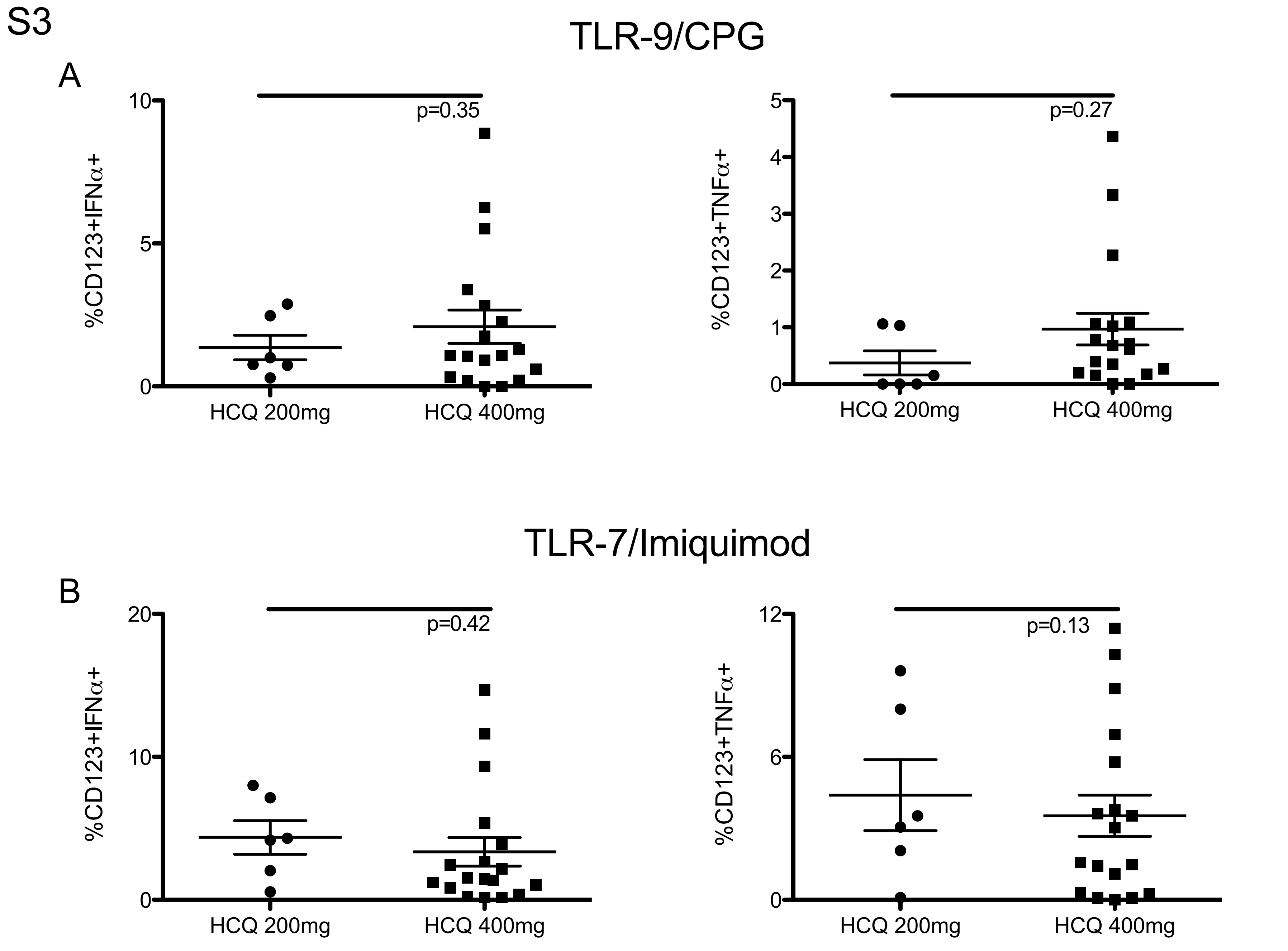

S3
TLR-9/CPG
A
%CD123+IFNα+
p=0.35
HCQ 200mg
HCQ 400mg
%CD123+TNFα+
p=0.27
HCQ 200mg
HCQ 400mg
TLR-7/Imiquimod
B
%CD123+IFNα+
p=0.42
HCQ 200mg
HCQ 400mg
%CD123+TNFα+
p=0.13
HCQ 200mg
HCQ 400mg
